# A case of Turcot’s syndrome type 1 with loss of immunoexpression of MSH6 in colon cancer and liver metastasis due to secondary somatic mutation in coding mononucleotide (C)8 tract: a case report

**DOI:** 10.1186/s12881-020-01079-x

**Published:** 2020-07-02

**Authors:** Shintaro Akabane, Takao Hinoi, Kiwamu Akagi, Hideki Yamamoto, Haruki Sada, Yosuke Shimizu, Wataru Shimizu, Takeshi Sudo, Takashi Onoe, Kohei Ishiyama, Takahisa Suzuki, Hirofumi Tazawa, Naoto Hadano, Toshihiro Misumi, Masato Kojima, Haruna Kubota, Daiki Taniyama, Kazuya Kuraoka, Hirotaka Tashiro

**Affiliations:** 1 Department of Surgery, National Hospital Organization, Kure Medical Center and Chugoku Cancer Center, 3-1, Aoyama-cho, Kure City, Hiroshima, 737-0023 Japan; 2 Department of Gastroenterological and Transplant Surgery, Graduate School of Biomedical & Health Sciences, Hiroshima University, 1-2-3 Kasumi, Minami-ku, Hiroshima, 734-8551 Japan; 3 Department of Clinical and Molecular Genetics, Hiroshima University Hospital, 1-2-3 Kasumi, Minami-ku, Hiroshima, 734-8551 Japan; 4 Department of Molecular Diagnosis and Cancer Prevention, Saitama Cancer Center, 3-1, Aoyama-cho, Kure City, Hiroshima, 737-0023 Japan; 5 Department of Clinical Laboratory, National Hospital Organization, Kure Medical Center and Chugoku Cancer Center, 3-1, Aoyama-cho, Kure City, Hiroshima, 737-0023 Japan; 6 Department of Diagnositic Pathology, National Hospital Organization, Kure Medical Center and Chugoku Cancer Center, 3-1, Aoyama-cho, Kure City, Hiroshima, 737-0023 Japan

**Keywords:** Lynch syndrome, Microsatellite instability, Mismatch repair gene, Somatic mutation, Turcot’s syndrome type 1

## Abstract

**Background:**

Lynch syndrome (LS), which is known as a hereditary cancer syndrome, is distinguished by microsatellite instability, represented by the altered number of repetitive sequences in the coding and/or non-coding region. Immunohistochemical staining (IHC) of DNA mismatch repair (MMR) proteins (e.g., MLH1, MSH2, MSH6, and PMS2) has been recognized as an useful technique for screening of LS. Previous study has shown that the assessment of IHC, however, requires specific caution due to variable staining patterns even without germline mutations in *MMR* genes.

**Case presentation:**

A 48-year-old man, who had been treated for anaplastic astrocytoma, was referred to our department for the precise examination of progressing anemia. Whole-body examination revealed two advanced carcinomas in descending colon and stomach. A hypo-vascular mass lesion was detected in liver as well. Pathological diagnosis (on surgical specimens) was poorly differentiated adenocarcinoma in descending colon, moderately differentiated tubular adenocarcinoma in stomach, and liver metastasis, which is possibly from colon. It was suspected that this case would be Turcot’s syndrome-type-1 due to its specific family history having two cases of colon cancer within the second relatives. Pathogenic frameshift mutations in codon 618 of *MLH1* gene was identified. Immunohistochemical analyses (IHC) demonstrated complete loss of MLH1 immuno-expression as well as of PMS2 except for those in brain tumor. Although frameshift mutation was not found in *MSH6* gene, histological expression of MSH6 was patchy in primary colon carcinoma and was completely lost in the metastatic site in liver. MSH6 expression in gastric carcinoma, a coincidental cancer in this case, was intact. An abnormal (C)8 region was identified by the cloned PCR of colon and liver tumors but not from gastric cancer. Frameshift mutation in a (C)8 tract in exon 5 of the *MSH6* gene was also detected in liver metastasis.

**Conclusion:**

This case supports a plausible mechanism, proposed by a previous literature, for the reduced expression of MSH6 in a somatic mutation manner, which might preferentially happen in colon cancer rather than in stomach carcinoma in MLH1/PMS2-deficient type of Turcot’s syndrome type 1.

## Background

Turcot’s syndrome type 1 is distinguished by brain tumor with LS-associated colon carcinoma or polyps, which is resulting from germline defects in mismatch repair genes [1, 2]. Immunohistochemical staining for DNA mismatch repair proteins, MLH1, MSH2, MSH6 and PMS2, has been reported as an useful method in screening colorectal cancer patients for LS [3]. LS-associated tumors arise as a result of inactivating mutations in DNA mismatch repair (MMR) system [4]. This system is essential in the repair of base mismatches, especially in repetitive base sequences known as microsatellites [5]. Cellular malignant transformation occurs due to the accumulation of these mutations [6].

Boland C et al. have shown that the coding region of *MSH6* gene have repeat sequences which might be affected by the defect of MMR genes [7].. In this case report, Turcot’s syndrome type 1 harboring the germline mutation of *MLH1* with loss of immunoexpression of MSH6 in colon cancer and liver metastasis due to secondary somatic mutation in coding mononucleotide tract in *MSH6* is presented. This phenomenon is compatible with the previous literature, that demonstrated the reduced expression of MSH6 in a somatic mutation manner, in colorectal carcinomas of MLH1/PMS2-deficient type of LS [8].

## Case presentation

**Fig. 1:**
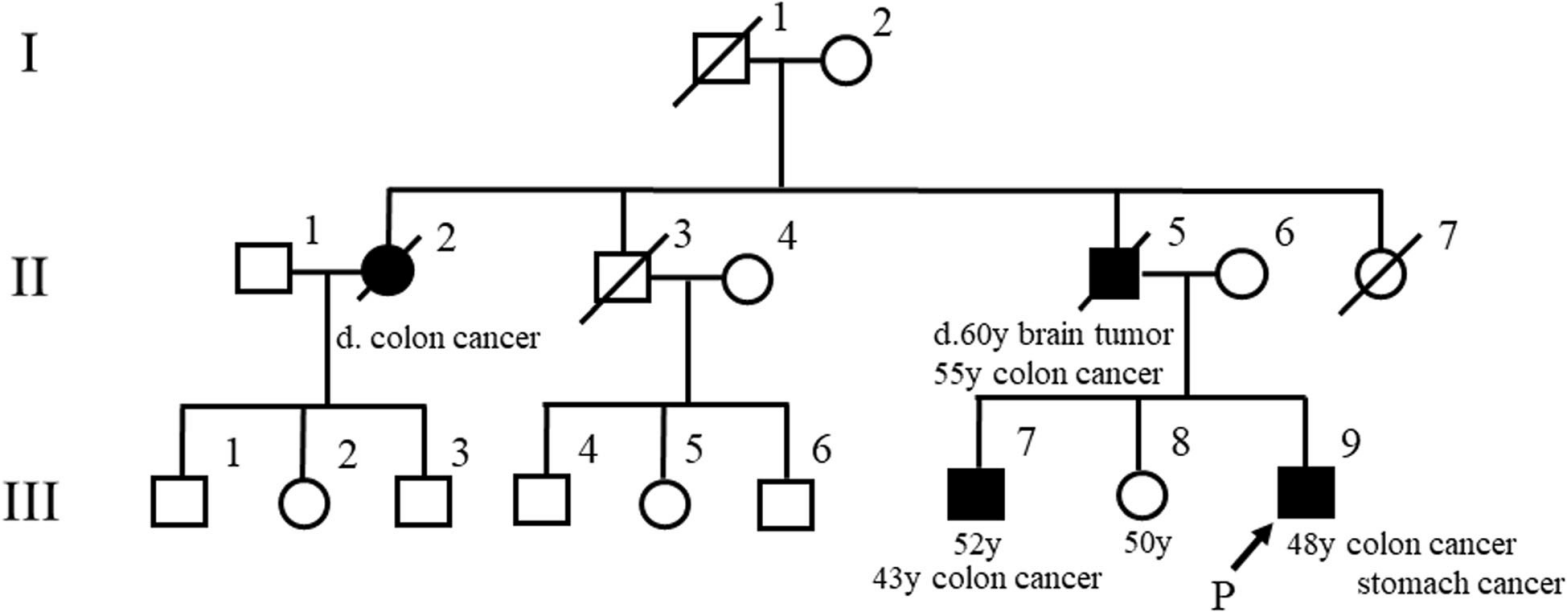
Pedigree of this patient’s family. The arrow indicates the proband and filled symbol indicates person with colorectal cancer

A 48-year-old man, who had a treatment history for anaplastic astrocytoma twice at the age of 39 and 46, was referred to our department for the inspection of progressing anemia. He was suspected as LS due to his specific family history by having two cases of colon cancer within his second relatives (Fig. 1). Whole-body examination revealed two foci of advanced stage of carcinoma in descending colon and stomach. A hypo-vascular mass in its size of 15 mm was also detected in S2 of liver. Pathological diagnosis using the biopsied samples and surgically resected specimens were poorly differentiated adenocarcinoma of descending colon, moderately differentiated adenocarcinoma of stomach, and poorly differentiated adenocarcinoma of the liver, suggesting the liver tumor would be metastasis from colon. 5-Fluorouracil-based adjuvant chemotherapy with the combination of oxaliplatin was selected for this case because this case was highly suspected as LS. During postoperative surveillance, repetitive polypectomy for colonic adenomas was performed, one of which was a 10 mm tubular adenoma with severe atypia in the ascending colon.

**Fig. 2:**
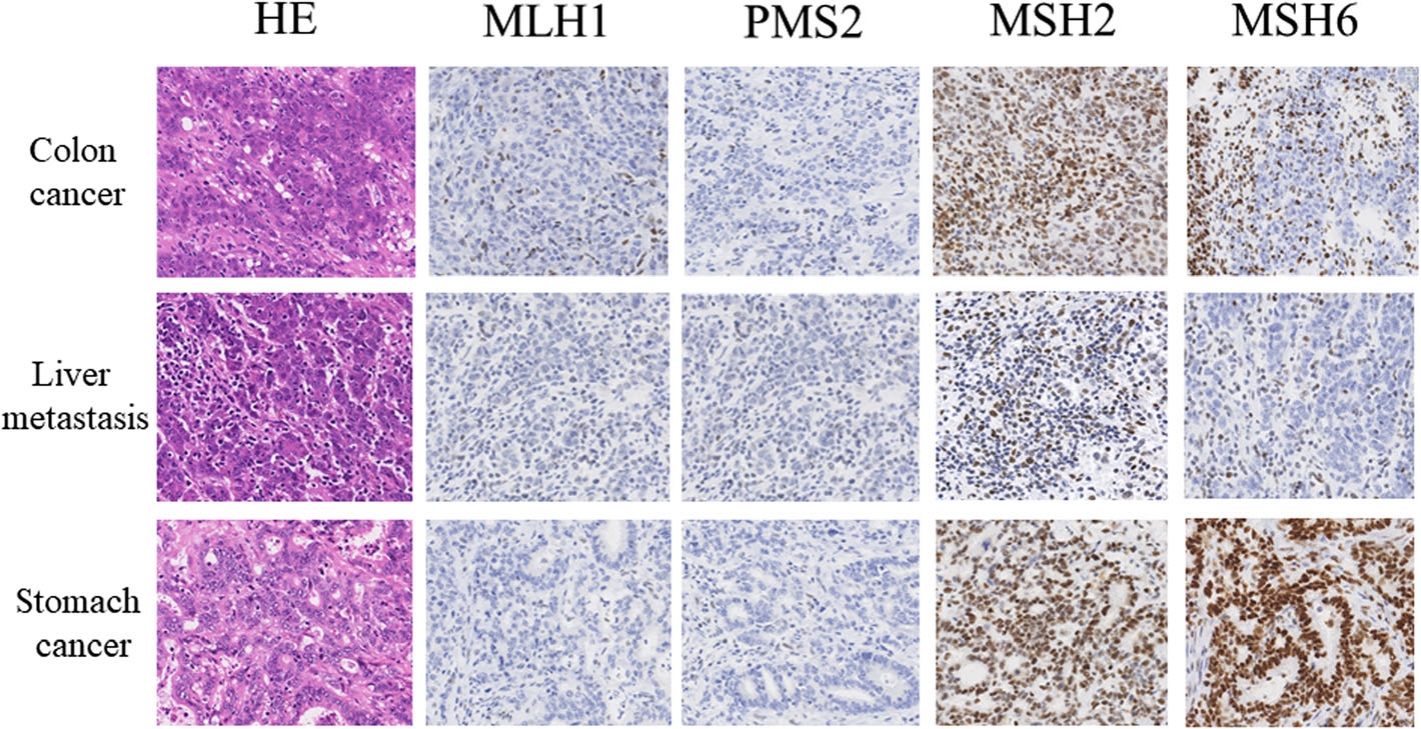
Histological examination and immunohistochemical staining for mismatch repair proteins of this patient’s tumors. HE staining of a colon tumor revealed a poorly differentiated adenocarcinoma with an increased intratumoral lymphocytes and neutrophils. By immunohistochemistry, the tumor is negative for MLH1 and PMS2 . This tumor has intact expression of MSH2. These glands show scanty staining for MSH6. HE staining of a stomach tumor revealed a moderately differentiated adenocarcinoma

**Fig. 3:**
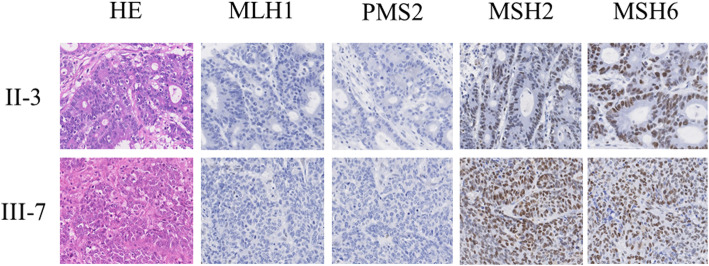
Immunohistochemical staining for mismatch repair proteins of the colon tumors in this patient’s family. By immunohistochemistry, the tumor of this patient’ s father; II-3 and brother; III-7 had an intact expression of MSH6. The rest of the mismatch repair proteins showed the same staining pattern of this patient; negative for MLH1 and PMS2, positive for MSH2

Detection of MSI was performed by Biomedical Laboratories, Inc. (Saitama, Japan) using a National Cancer Institute–recommended panel of microsatellite markers (BAT25, BAT26, D2S123, D5S346, and D17S250) and additional markers (D2S136, D3S1067, TP53, D18S51). This analysis showed a positive result for eight markers (except D5S346), indicating a high frequency of MSI (MSI-high). Immunohistochemistry (IHC) for MMR proteins of this patient’s tumors were also performed. Monoclonal antibodies (MLH1: mouse monoclonal, clone ES05, MSH2: mouse monoclonal, clone FE11, MSH6: rabbit monoclonal, clone EP49, PMS2: rabbit monoclonal, clone EP51 {DAKO, Denmark}) were used for the present study. The IHCs of the descending colon cancer demonstrated abnormal nuclear staining of scanty MSH6, concurrent loss of MLH1 and PMS2, whereas the staining for MSH2 was positive. On the other hand, IHCs of the liver metastasis showed complete loss of MSH6, MLH1, PMS2 and the staining for MSH2 was positive. IHCs of the stomach cancer revealed negative staining of MLH1 and PMS2, whereas the staining for MSH2 and MSH6 were positive. These results are summarized in Fig. 2. Further investigation in IHCs of the colon cancer specimens from his father and brother were performed. Both of them showed the same staining pattern, as of this patient’s stomach cancer (Fig. 3).

**Fig. 4:**
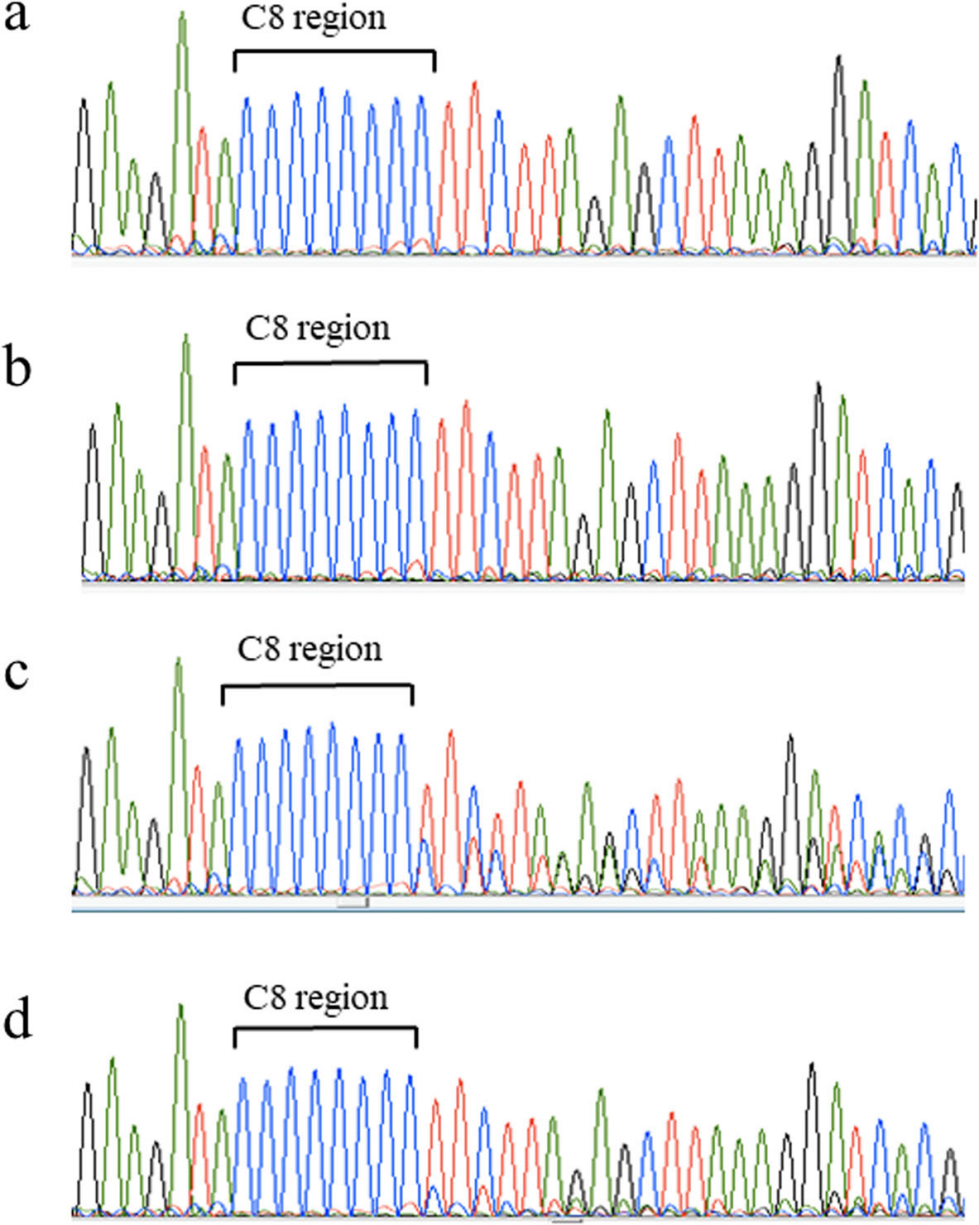
Electropherograms depicting the coding region microsatellite (C)8 in exon 5 of MSH6 from this patient’s tumors. An intact (C)8 region was shown in a normal mucosa of the colon (**a**) and stomach cancer (**b**). An abnormal (C)8 region was detected in the colon cancer (**c**) and the liver metastasis (**d**)

A gene mutation of *MLH1*was suspected in this family line, because all of the colon cancers demonstrated complete defects of MLH1 and PMS2. Genetic analysis for mismatch repair genes, *MLH1, MSH2, MSH6, PMS2, MSH3, PMS1, EPCAM, MLH3, APC, MUTYH, POLD1, POLE, TP53, AXIN2* and *BMPR1A* was conducted. This genetic testing revealed a frame shift mutation in codon 618 (c. 1852-1854delAAG / p.Lys618del) of MLH1. This mutation is registered on the database of InSiGHT as class 5 and as pathogenic in Clin Var [9, 10]. To investigate the mechanism of the scanty staining pattern of MSH6 in colorectal carcinoma, analysis of the coding region microsatellite (C)_8_ in exon 5 of MSH6 in this patient’s colon cancer, stomach cancer, and liver metastasis was performed. An intact (C)8 region was shown in a normal tissue of the colon and stomach cancer. An abnormal (C)8 region was seen in the colon cancer and the liver metastasis (Fig. 4). The distribution of the mutant alleles in the (C)_8_ tract was further analyzed by cloning and sequencing analysis. Deletion mutation (−1wt) and insertion mutation (wt + 1) were detected in the liver metastasis. Representative electropherograms of wild type, −1wt and wt + 1 clones are shown in Supplementary Fig. 1.

## Discussion and conclusions

The role of MMR protein during DNA replication is to retain genomic stability by correcting the base mismatches [11]. We have been investigating the molecular alterations of the deficient MMR colon cancer and previously reported about the subset of poorly differentiated colon carcinoma with microsatellite instability harboring less expression of CDX2 [12]. Although CDX2 has a microsatellite (G)7 sequence tract in its coding region, our previous analysis showed that its marked reduction was a result of dominant-negative pathways regulating CDX2 transcription [13]. In contrast, in the present case, the reduced expression of MSH6 with a microsatellite (C)8 region is likely due to a somatic mutation caused by loss of an MMR component.

As shown in this case, this patient was preoperatively suspected of Lynch syndrome from his family history. Subsequent analysis of IHC for MMR proteins demonstrated MLH1/MSH6 deficiency in colon cancer and liver metastasis, and MLH1 deficiency in gastric cancer. On the other hand, colon cancer specimens of his father and brother showed only MLH1 deficiency. This family line was indicated to have *MLH1* gene mutation and genetic testing revealed a frame shift mutation in *MLH1* gene. Therefore, the reduced expression of MSH6 in colon cancer and liver metastasis was suspected as somatic mutation manner.

It has been shown that the coding region of *MSH6* gene have repeat sequences which might be target gene for instability [14]. The coding region microsatellite (C)_8_ in exon 5 of MSH6 in the colon cancer and liver metastasis were analysed by DNA sequencing and showed mutation. The rational story is that microsatellite unstable colon cancer cell due to the *MLH1* gene mutated condition metastasized to the liver and occurred somatic mutation into another type of allele in both tumor sites. The origin of metastatic liver tumor had already been diagnosed by morphological analysis, however, the results of DNA sequence also proved that the liver metastasis was not derived from stomach cancer, because the (C)_8_ region in exon 5 of MSH6 was wild type in the stomach cancer. We speculate that the reduced expression of MSH6 is a passenger mutation rather than a driver mutation in carcinogenesis in this case. However, it is possible that MSH6 deficiency due to the *MLH1* gene mutation played a partial role in metastatic disease formation, which is consistent with the difference in MSH6 expression between the metastatic region and the primary site. Further investigations in similar cases comparing the primary tumor site and metastatic regions might provide a biological implication on whether secondary mutations in MSH6 contribute to carcinogenesis.

There must be a possible change of recognition potency of MSH6 antibody against MSH6 with the frameshift mutation in the repetitive mononucleotide tract. It has been reported that the truncated polypeptide due to 1 bp deletion or insertion at codon 1116 of MSH6 coding region was unstable and was not detected by the in vitro protein expression analysis [15]. We presume that the similar mechanism resulted in the loss of MSH6 immunoexpression. There have been some reports about the frequency of somatic frameshift mutations of the *MSH6* gene induced by microsatellite instability. Duval A. et al. reported in their review article that somatic frameshift mutation of MSH6 was detected in more than 30% of colorectal cancers [14]. On the other hand, Shia J. et al. have shown the frequency of scanty staining of MSH6 was about 2% in colorectal carcinoma cases that meet the Revised Bethesda Guidelines [8], and Rondell et al. also showed the mutation ratio was less than 1% of colorectal cancer from the immunohistochemical analysis in a larger population [16].

Although the interpretation of IHC patterns require cooperation with pathologists, it has some advantages such as its inexpensiveness and convenience for direct detection of altered MMR genes, and its potency for visual evaluation by anyone [3]. However, there are some pitfalls of the interpretation of the results of IHC. As one of which was described in this case, the failure of MLH1 function associated with the somatic mutation of MSH6 coding region. Various staining patterns of MMR proteins, especially in MSH6, need to be recognized to prevent misinterpretation of IHC results, which do not necessarily represent the germline mutations.

This patient had been treated for anaplastic astrocytoma, therefore, this case was appeared to be related to Turcot syndrome type 1 which is characterized by glioblastoma and colorectal cancer or polyps associated with LS [1, 2]. However, the IHCs of the specimen of brain tumor showed intact staining of all MMR proteins (Supplementary Fig. 2). The staining pattern was discrepant with the previous report that the existence of germline mutation may influence the carcinogenesis of glioblastoma and colorectal neoplasm in Turcot’s syndrome patients [9]. It is also reported that chromosomal instability play an important role in the tumorigenesis of sporadic glioblastoma multiform [17], the brain tumor of this patient was considered to be a sporadic case or derived from another mechanism other than the deficiency of MMR system.

MMR status is meaningful in selecting pharmacological strategy, because deficient MMR cancers may be resistant to 5-FU based chemotherapy [18, 19]. Adjuvant chemotherapy using oxaliplatin has been commonly recognized as standard regimen for patients with node-positive colon cancer and advanced stage of gastric cancer regardless of the mismatch repair status. Some clinical trials showed more beneficial outcomes of the postoperative adjuvant FOLFOX treatment for patients with loss of MMR than those without MMR deficiency [20, 21]. Additionally, a cohort study proved the preference of oxaliplatin-using postoperative treatment for the prognosis of stage III colon cancer with MSI-high status [11, 22]. Furthermore, recent study has shown that MMR deficient carcinoma patients were shown to have better prognosis by immune checkpoint blockade [23].

This report indicates the underlying mechanism for the patchy expression pattern of MSH6 and it was proved by the frameshift mutation of the repetitive C8 region. This case supports a theoretical mechanism for the reduced expression of MSH6 due to a somatic mutation in the coding region that might arise in colon cancer of MLH1/PMS2-deficient type of LS.

## Supplementary information

**Additional file 1: Figure S1.** Electropherograms of the cloned PCR products from liver metastasis; the wild-type sequence (a); deletion of 1 C, −1wt (b); insertion of 1 C, wt + 1 (c).

**Additional file 2: Figure S2.** Histological examination and immunohistochemical staining for mismatch repair proteins of this patient’s brain tumors, at the age of 39 (a) and 46 (b) HE staining of the brain tumors showed anaplastic astrocytoma. The tumors showed intact staining for the MMR proteins.

## Data Availability

The variant reported in here is available in the InSiGHT DNA Variant Database repository, (http://www.insight-group.org) and in the Clinvar repository, [with accession ID: VCV000017080.7] (https://www.ncbi.nlm.nih.gov/clinvar/variation/17080/). The raw datasets generated and/or analysed during the current study are not publicly available in order to protect participant confidentiality.

## Abbreviations

LS: Lynch syndrome
MMR: Mismatch repair
DNA: Deoxyribonucleic acid
PET: Positron emission tomography
CT: Computed tomography
FDG: Fluorodeoxyglucose
SUV: Standardized uptake value
MSI: Microsatellite Instability
IHC: Immunohistochemistry
H&E: Hematoxylin and eosin
