# Supplementary figures and images for: A case of Turcot’s syndrome type 1 with loss of immunoexpression of MSH6 in colon cancer and liver metastasis due to secondary somatic mutation in coding mononucleotide (C)8 tract: a case report
Source: BMC Med Genet. 2020 Jul 2;21:141. doi: 10.1186/s12881-020-01079-x (PMC7345515; doi:10.1186/s12881-020-01079-x)

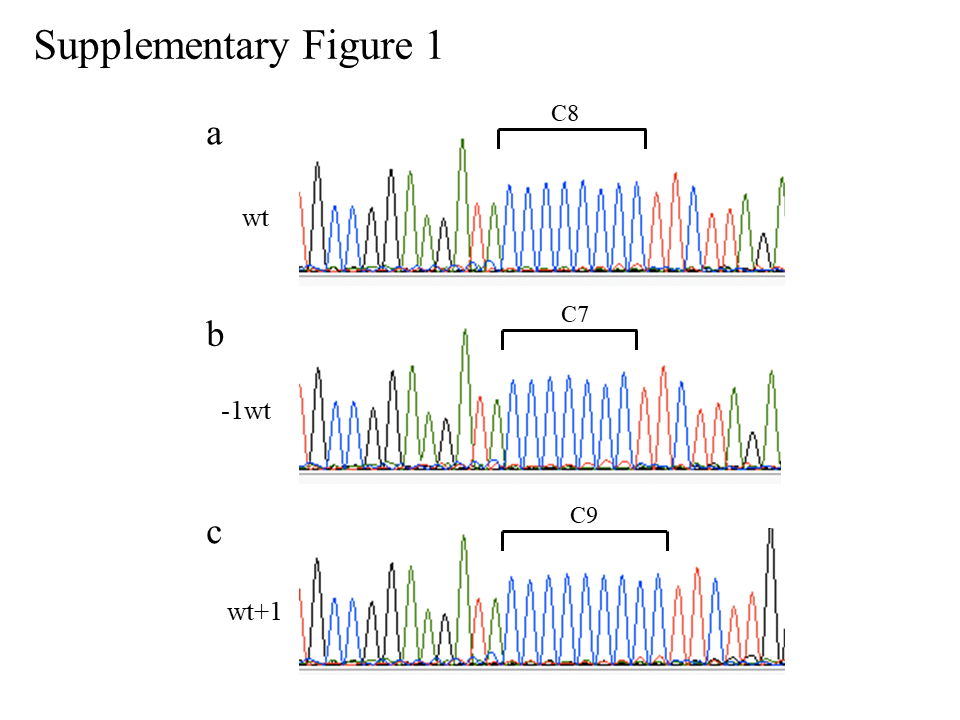

Supplement: Supplementary file 1 — Additional file 1: Figure S1. Electropherograms of the cloned PCR products from liver metastasis; the wild-type sequence (a); deletion of 1 C, −1wt (b); insertion of 1 C, wt + 1 (c). [file 12881_2020_1079_MOESM1_ESM.tif]

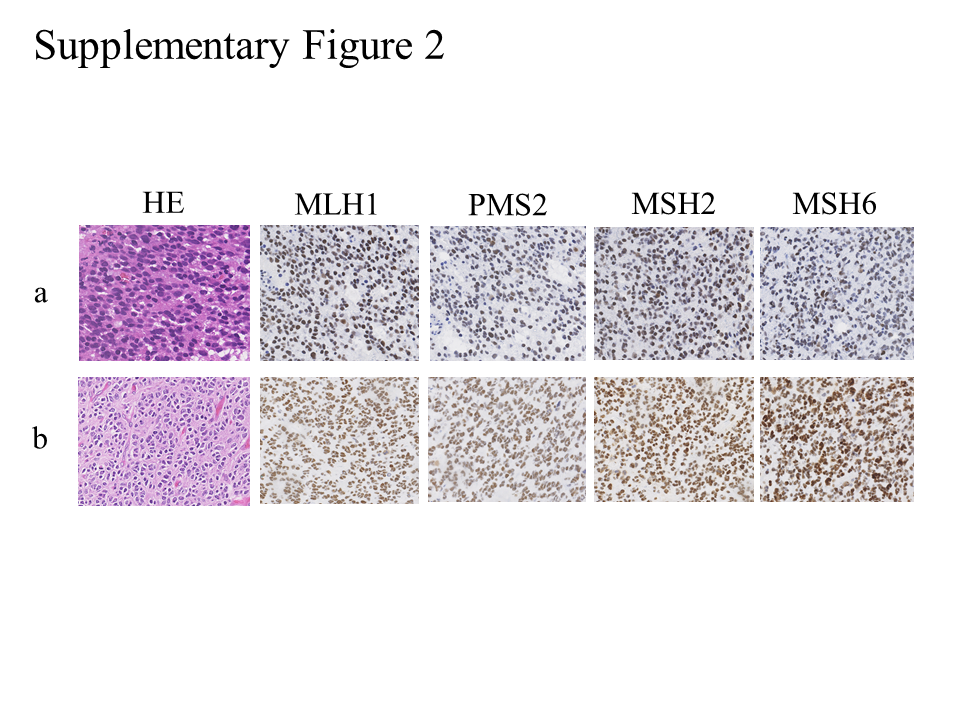

Supplement: Supplementary file 2 — Additional file 2: Figure S2. Histological examination and immunohistochemical staining for mismatch repair proteins of this patient’s brain tumors, at the age of 39 (a) and 46 (b) HE staining of the brain tumors showed anaplastic astrocytoma. The tumors showed intact staining for the MMR proteins. [file 12881_2020_1079_MOESM2_ESM.tif]
